# Annual Dinner of the Atlanta Society of Medicine

**Published:** 1886-07

**Authors:** 


### ANNUAL DINNER OF THE ATLANTA SOCIETY OF MEDICINE.

The first annual dinner of the Atlanta Society of Medicine was given on the evening of June the 17th. Beside a large number of local physicians present, Drs. Henry F. Campbell, of Augusta, and Robert Battey, of Rome, honorary members of the Society, were present. There were a number of good speeches made. Dr. Campbell, in speaking of the advantages of organization, thought that organization was the idea that prompted the formation of the Medical Society. By it the cross-road doctor, the hamlet doctor, was able to come and sit side by side with the great lights of the profession and discuss with them the subjects of common interest. Out of these small societies the organization of the entire profession was effected, and in America there resulted that grand body, the American Medical Association. Lately there has been some bad feeling. Many persons have had differences, but the disappointment will not be great and the American Medical Association will remain the bulwark and defense of the American medical profession. Organization never fails to do its excellent work.

Dr. Olmsted made some good hits on the subject of medical ethics. He said medical ethics was a matter for practice, not preaching ; that a gentleman was never made by any code of ethics, and that there were many ways in which a physician could act ungentlemanly, at the same time not violating strictly the written code. He thought it behooved the profession to make vital what is apt to become too often a philosophic abstraction.

Dr. Taliaferro spoke of the evil results of higher education of women. He denounced in unmeasured terms the "pressure" system of education in our fashionable boarding-schools and female colleges. He said that the present birth-rate, when compared to that of olden times, shows a degeneracy of the constitution of women of the present day. He thought that physical culture was a first consideration, and that, as a girl approached womanhood, she needed all her energy for the development of certain organs and the exercise of their natural physiological functions. If, at this period, her energy is devoted to the development of her intellect, the consequences are, imperfectly developed ovaries, dysmenorrhœa, hysteria, physical impairment and perhaps insanity. He thought that it was the duty of the physician to make his influence felt upon this question among the families who were under his charge. He further claimed that the girl who wins the prize is an invalid, the girl who stands first in the class is an invalid, and that highly intellectual women are not fit for matrimony—that higher education is damaging to women, damaging to humanity.

Doctor Battey made some interesting remarks on the subject of Laparotomy Epidemic. He said, "there is an epidemic," and he wished to sound the alarm. He thought that we were going too fast, that we ought to be more conservative in our abdominal surgery. Speaking more particularly, he said he thought that the entire extirpation of the uterus for malignant disease was an exceedingly questionable procedure, and that the operation that bore his name was also abused; it was done too often. He then alluded to his claims as the originator and discoverer of the so-called Battey's operation, that he was content to await the verdict of his professional brethren when they have canvassed the matter. He was startled at the assertion recently made by Mr. Lawson Tait, of Birmingham, in the Philadelphia *News*, that his original case was fatal, while his (Tait's) own was successful, while the file of the *British Medical Journal* of 1879 shows that Tait's operation was fatal in its result. Dr. Battey then referred to his first case, in which he operated in August, 1872, with success. The same was reported in September, in THE ATLANTA MEDICAL AND SURGICAL JOURNAL, and in April following was brought before the State Medical Society at Atlanta and discussed *in extenso*. There were two other claimants in the field besides himself. Hegar, of Freiburg, claims to have performed the operation July 17th, 1872, one month prior to his own (Battey's) operation. Hegar admits that his patient died, and that he did not repeat the operation until "the battle was fought and won in America." In 1875, he performed several operations in Kentucky, which were recorded in THE ATLANTA MEDICAL AND SURGICAL JOURNAL. He was requested by a German physician, of Louisville, to send on copies of THE ATLANTA MEDICAL AND SURGICAL JOURNAL for distribution in Germany. He did not know whether Hegar received a copy or not, but it was a matter of history that Hegar did not repeat the operation until 1877 or 1878. As to the claim of Mr. Lawson Tait, Dr. Battey made remarks which were listened to with great attention. He spoke of the report of Mr. Tait in the *British Medical Journal* in 1879, in which it was claimed that the operation was done three times, each time with fatal results. He then referred to a visit which he made to Mr. Tait, in 1881, in which that gentleman said that he had made a mistake in his report in the *British Medical Journal;* that his case, in 1875, did not die, but turned out to be a brilliant case. "This he discovered lately. There was no such writhing in his soul as there was in mine when the operation was performed, or he would not have waited seven years to ascertain if the patient lived or died." Dr. Battey said that he was astonished at the statement and made no reply at the time. He never expected that Mr. Tait would have the audacity to appear in print as he did. "For my part," said Dr. Battey, "I never would have opened my mouth after seven years."

Drs. Todd, Gaston and Hardon also responded to regular toasts of the evening: Dr. Todd, on the "Medical Profession;" Dr. Gaston, "The Atlanta Society of Medicine," and Dr. Hardon, "Poetry and Physic." The speeches were highly entertaining and contributed much to the enjoyment of the occasion. The affair was a grand success.

### ITEMS.

DR. J. P. TAYLOR, of Haralson, Ga., passed through Atlanta a few days ago on a visit to Roanoke, Va.

DR. J. G. HOPKINS has returned to Thomasville and entered into the practice of medicine with his father, Dr. T. S. Hopkins.

DR. T. J. WORD, of this city, has recently purchased a $20,000 house on Peachtree and has removed from Whitehall, where he formerly resided, to his new home.

MR. P. C. MAGNUS, of this city, was elected First Vice-President of the State Pharmaceutical Association at the recent meeting of that body in Savannah.

DR. J. M. HULL, of Augusta, who was prevented from attending the recent meeting of the State Association by sickness, has, we are glad to state, recovered.

THE National Eclectic Medical Association was in session in this city June 16th, 17th and 18th. There were one hundred and eighty delegates in attendance.

DR. J. McF. GASTON, of this city, has been invited to contribute a paper on surgical relations of the gall-bladder to the duct, to the next meeting of the British Medical Association.

DR. VIRGIL O. HARDON, of this city, has been appointed lecturer on operative gynæcology in the Southern Medical College. The college could not have made a better selection.

DR. H. MCHATTON, of Macon, one of the most prominent physicians in the State, passed through Atlanta a few weeks ago on a pleasure trip to the mountains of Western North Carolina.

DR. HUNTER P. COOPER, late of the Presbyterian Eye and Ear Hospital, New York, has been elected adjunct professor of chemistry in the Atlanta Medical College. We congratulate the faculty on securing the services of one so competent.

THE Canada *Medical Record* for May did us the honor to reproduce our translation of M. Pajot's remarks upon Alexander's operation, which appeared in the April number of THE JOURNAL. We presume that the failure to give us credit for the same was an oversight.

CINCINNATI COLLEGE OF MEDICINE AND SURGERY.—Elsewhere will be found the card of this college. It is growing in popular favor every year. The next session will begin September 13th with a full faculty. Dr. R. C. Stockton Reid, 164 George street, Cincinnati, is the Dean.

WE had a pleasant call a few days since from Dr. J. E. English, representing the well known house of Seabury & Johnson, New York, manufacturers of surgical plasters, antiseptic dressings and absorbents. Dr. English is justly popular and never fails to meet with a warm welcome whenever he calls on our physicians.

A NEW JOURNAL.—We have received the first number of the *Alabama Medical and Surgical Journal*, edited by Drs. John D. S. Davis and W. E. B. Davis, of Birmingham. We congratulate the editors upon its handsome appearance, and wish for it all the success it so justly deserves.

THE TULANE UNIVERSITY.—This old institution, under a new name, is the best equipped medical institution in the South for giving clinical instruction. The professors are given by law the use of the great Charity Hospital as a school of practical instruction, and students are admitted without payment of any hospital fees. The hospital contains seven hundred beds, and is kept constantly full of patients representing every class of disease. Over twenty thousand patients are treated annually. The practical advantages offered by the Tulane University are unsurpassed by any medical college anywhere.

THE CENTURY DICTIONARY.—For the past five years the Century Company has been engaged in preparing a dictionary of the English language, of which Professor William D. Whitney, of Yale College, is editor-in-chief—the purpose being to make a more comprehensive work than has yet appeared in popular form, to include, in addition to a very full collection of individual words in all departments of the language, all technical phrases, not self-explaining, in law, the mechanical arts, the sciences, etc. Indeed, it is designed to make this dictionary so complete in its definitions of all branches of science and art that even the specialist will need nothing further. The number of "new" words in many of these departments is said to be surprisingly great. The dictionary will have also a remarkably complete system of cross-references, and will embody in itself a dictionary of synonyms which will add greatly to its value.

PROF. N. GRAY BARTLETT, favorably known from his long connection with Chicago College of Pharmacy, will re-occupy the chair of pharmacy the coming year. We would note in this connection that the Chicago College of Pharmacy is an old and tried institution—antedating the war of the rebellion—sustained and supported in its earliest years by the private contributions of Chicago's enterprising druggists. The present college building was located and planned expressly for the purposes of pharmaceutical education. The location is accessible and peculiarly favorable for health, situate near the shore of Lake Michigan, away from the noise and clatter of central trade. The lecture hall has the advantage of a ground-floor location, with easy exit in case of fire, and will accommodate 600 students with chairs, and has perfect light and ventilation. Laboratories are large and well-equipped in every department.

WHERE TO SPEND THE SUMMER.—There is not a more beautiful or pleasant country on the globe to spend the hot summer months than the mountain region of Western North Carolina. The scenery is unsurpassed in beauty by any in the world, while the climate and water is all that could be desired by any one. This beautiful country is reached by the Piedmont Air-Line, one of the best-managed and equipped railroads in the South, forming the shortest and most direct route between the South and North. It operates and controls the Western North Carolina Railroad, which penetrates every part of this grand and beautiful country.

LACTOPEPTINE FOR DYSPEPSIA.—Dr. Livezey writes (*The Medical Summary*): "While wintering in Florida I met with my annual patient, a young lady of twenty-eight, from Chicago, who was sent hither three or four years ago in order to pass out into the 'spirit land' comfortably, who, now being troubled with poor appetite, slight but distressing nausea, great debility, irregular menstruation, excessive cardiac action on the least exertion, etc., I ordered 1 oz. bottle of lactopeptine of the N. Y. Pharmacal Association's manufacture, and she improved at once. Soon after, she met a lady friend, who told her she ought to take lactopeptine, stating what wonders it had done her, who was troubled 'just the same way' (of course). 'Why, bless me,' said my patient, 'that is just what my doctor prescribed for me, and I am doing nicely.' By the time she finished the small vial, she declared she never felt better in her life, her appetite being regular and everything O. K. She has taken since lactopeptine, elixir, calisaya, iron and bismuth with excellent results."

IN the Texas *Courier-Record*, of April, 1886, Dr. A. W. Fly, in speaking of "division of the stricture and ignoring the use of sound in after treatment," alleges that it is "not practiced by any other surgeon except Dr. Gaston, of Atlanta, Ga." Our readers will remember the full description of this operation by Dr. Gaston in the April number of THE JOURNAL for 1884, referring to his first observation ten years previously of this procedure in the hands of Dr. V. Saboia, the professor of surgery in the Imperial Medical Academy of Brazil. He insisted that when a stricture is completely cut through, there was no indication for the use of any means of dilatation, while introduction of the bougie or catheter was invariably a source of trouble.

BROMIDIA.—J. Lindsay Porteous, M. D., F. R. C. S. M. R. C. P. ED., in the April number of the Edinburgh *Medical Journal*, says: Of late there has been a great influx of new drugs, some of great value, others of little or no use. Where a medical man has an extensive practice, consisting of rural and urban patients, he has ample opportunity of testing the effects of drugs, as the varieties of disease that come under his notice are great; and although his means of watching the actions of drugs are not so good as in hospital practice, yet a good deal can be done if he cares to take a little trouble to "take notes." The following is one which has been used for some time by my colleague (Dr. Proudfoot) and myself, and I give the results: About eighteen months ago a friend of mine from America told me of the wonderful effects of a medicine, much used in the States, called bromidia. According to the makers, it is composed of chloral hydrate, 15 gr.; potassium bromide, 15 gr.; extract of cannabis indica, $\frac{1}{8}$ gr. and extract of hyoscyamus $\frac{1}{8}$ gr. I obtained some, and have ordered it regularly for over a year, and have found it excellent in the pain of rheumatism, pneumonia, and cancer; also in the sleeplessness of scarlatina and alcoholism. It has never failed me in procuring sleep, without the disagreeable dreams and after-effects of opium. The dose is ʒss. to ʒj. every hour till sleep is procured. I have also found it of much service in cases of tonsillitis, used as a gargle with glycerine and carbolic acid.

DR. W. F. WESTMORELAND, Jr., after a stay of several months in New York, has returned to the city.

THE circulation of the New York *World* is 170,000 daily, and over 230,000 on Sunday.

CARNRICK'S SOLUBLE FOOD.—Dr. C. F. Dennty, of St. Paul *Northwestern Lancet*, June 1st, 1886), writes: Not long since I had brought to me a child of six months, suffering from the following symptoms: constipation, at times irregular action of bowels, regurgitation of food and an asthmatic cough. Its mouth was full of thrush sores, and its appearance one of poor nourishment. It had been given a number of Infants' Foods in vain, one of which I prescribed myself. By means of mild medication, directed towards the cough and stomach, something was accomplished. Finally I gave "Carnrick's Soluble Food," and had the satisfaction of having it retained, and at last accounts the child was doing nicely. I am inclined to think this food is worthy of attention on the part of the profession. It recommends itself, in that it contains caseine, rendered soluble by pancreatine, starch converted into dextrine and maltose. Hence it requires but little preparation, and that is so simple, mistakes cannot occur. It requires no addition of milk. It has the advantages and none of the disadvantages of the many foods now in the market, and forms a nearly physiological substitute for mother's milk.

BOWDEN LITHIA SPRING, near this city, is being improved and is developing into a first-class health resort. It promises in the near future to become the Saratoga of the South.

CRIMINAL ABORTION.—It is rumored that a lady (?) of Birmingham, who contemplates matrimony at an early date, had an abortion committed upon her about the 20th May. The infamous scoundrel and she-devil who are guilty of this heinous crime will be remembered and watched. They have been cunning and sly, but our promise for it, if they are not careful, law and public sentiment enough yet will be stirred up to get a verdict of guilty. May something be done to eradicate this wholesale slaughter of the helpless!—*Alabama Med. and Surg. Journal.*

ON going to press we are in receipt of the report of the proceedings of the Fifteenth Congress of German Surgeons, held at Berlin, April 7th, 8th, 9th, 10th, 1886. The report appears as a supplement to the current number of the *Centralblatt fur Chirurgie.* The German Congress of Surgeons is composed of men of the highest ranks in the profession, and the report of the work done at the last session contains many valuable contributions to surgery.

METEOROLOGICAL SUMMARY FOR MAY, 1886, STATION, ATLANTA, GA.

| | |
|---|---|
| Mean Temperature, | 68.0 |
| Highest Temperature, May 13th, | 90.0 |
| Lowest Temperature, May 1st, | 44.0 |
| Monthly Range of Temperature, | 46.0 |
| Greatest Daily Range of Temperature, | 28.0 |
| Least Daily Range of Temperature, | 9.0 |
| Mean Daily Range of Temperature, | 20 0 |
| Prevailing Direction of Wind, | S. W. |
| Highest Velocity of Wind and Direction, | 32 Miles, W. |
| Total Precipitation. | 6.21 inches. |
| Number of Days on which .01 inch or more of Rain fell | 9.0 |
| Number of Foggy Days | .0 |
| " " Clear " | 16.0 |
| " " Fair " | 13.0 |
| " " Cloudy " | 2.0 |
| Dates of Thunder-storms, | 6, 7, 15, 18, 21, 22, 24, 29, 30 |

